# A quantitative evaluation of the extent of fluralaner uptake by ticks (*Ixodes ricinus*, *Ixodes scapularis*) in fluralaner (Bravecto^TM^) treated vs. untreated dogs using the parameters tick weight and coxal index

**DOI:** 10.1186/s13071-015-0963-6

**Published:** 2015-06-30

**Authors:** Heike Williams, Janina Demeler, Janina Taenzler, Rainer K.A. Roepke, Eva Zschiesche, Anja R. Heckeroth

**Affiliations:** MSD Animal Health Innovation GmbH, Research Antiparasitics, Zur Propstei, 55270 Schwabenheim, Germany; Free University of Berlin, Institute of Parasitology and Tropical Veterinary Medicine, Robert-von-Ostertag-Str. 2, 14163 Berlin, Germany

**Keywords:** Bravecto^TM^ chewable tablets, Fluralaner, Dog, Tick weight, Coxal index, *Ixodes ricinus*, *Ixodes scapularis*, Efficacy

## Abstract

**Background:**

Fluralaner is a new antiparasitic drug that was recently introduced as Bravecto^TM^ chewable tablets for the treatment of tick and flea infestations in dogs. Most marketed tick products exert their effect via topical application and contact exposure to the parasite. In contrast, Bravecto^TM^ delivers its acaricidal activity through systemic exposure. Tick exposure to fluralaner occurs after attachment to orally treated dogs, which induces a tick-killing effect within 12 h. The fast onset of killing lasts over the entire treatment interval (12 weeks) and suggests that only marginal uptake by ticks is required to induce efficacy. Three laboratory studies were conducted to quantify the extent of uptake by comparison of ticks’ weight and coxal index obtained from Bravecto^TM^-treated and negative-control dogs.

**Methods:**

Three studies were conducted using experimental tick infestation with either *Ixodes ricinus* or *Ixodes scapularis* after oral administration of fluralaner to dogs. All studies included a treated (Bravecto^TM^ chewable tablets, MSD Animal Health) and a negative control group. Each study had a similar design for assessing vitality and weighing of ticks collected from dogs of both groups. Additionally, in one study the coxal index (*I. ricinus*) was calculated as a ratio of tick’s ventral coxal gap and dorsal width of scutum. Tick weight data and coxal indices from Bravecto^TM^-treated and negative-control groups were compared via statistical analysis.

**Results:**

Ticks collected from Bravecto^TM^-treated dogs weighed significantly less (*p* ≤ 0.0108) than ticks collected from negative-control dogs, and their coxal index was also significantly lower (*p* < 0.0001). The difference in tick weights was demonstrated irrespective of the tick species investigated (*I. ricinus*, *I. scapularis*). At some assessments the mean tick weights of Bravecto^TM^-treated dogs were significantly lower than those of unfed pre-infestation (baseline) ticks. The demonstrated tick-killing efficacy was in the range of 94.6 – 100 %.

**Conclusions:**

Tick weights and coxal indices confirm that a minimal uptake results in a sufficient exposure of ticks to fluralaner (Bravecto^TM^) and consequently in a potent acaricidal effect.

## Background

Numerous options for tick control in companion animals are known and used by veterinarians and pet owners. Existing commercial products are usually topical treatments such as spot-ons, sprays or collars, which have a tick-killing and/or repellent effect (also known as ‘knock down’ effect in pyrethroids [1]) via contact exposure of ticks to the treated animal [1, 2], e.g. a dog. An alternative for tick control in dogs that provides a different mechanism of exposure is the systemic treatment of ticks and fleas delivered through a chewable tablet (Bravecto^TM^, MSD Animal Health [3]; NexGard^TM^, Merial [4]). This option of tick control exerts its effect when ticks attach to the host and are exposed to a drug from the isoxazoline chemical class (e.g. fluralaner [3, 5]; afoxolaner [4]) that was absorbed in the host’s gut and distributed through the blood circulation. An uptake of the medicated tissue fluid by the parasite results in a potent tick-killing effect and also provides insecticidal effects against fleas [3, 4, 6, 7].

Fluralaner (Bravecto^TM^, MSD Animal Health) offers veterinarians and dog owners long-lasting tick and flea control after a single oral administration (12 weeks [3, 6–10]), a fast onset of parasite-killing effect (ticks: 12 h [8]; fleas: 8 h, [6]), prevention of flea reproduction in the dogs’ living environment [7] and a favorable safety profile [11–13] even in MDR1(−/−) Collies [14]. Fluralaner also offers ease of administration, due to its high success rate of being taken voluntarily when offered as a chewable tablet to dogs [10].

Irrespective of their mode of action, the ultimate aim of all tick control products is to provide prevention of tick infestation, ideally before engorgement occurs and tick-borne pathogens are transmitted. Two recently introduced systemic products have demonstrated successful tick control after a single oral administration to dogs for 4 weeks (NexGard^TM^ [4]) or for 12 weeks (Bravecto^TM^ [3]). However, pet owners may perceive the systemic nature of a product as a drawback because ticks need to attach to enable the product to become effective.

Fluralaner’s fast onset of action [8] provides strong evidence that the uptake required for efficacy is extremely small. The aim of the study described in this paper was to investigate the extent of uptake by means of comparing weight and coxal index of ticks obtained from fluralaner- (Bravecto^TM^) treated and control dogs. A significant reduction in these measurements between ticks from untreated control dogs compared to fluralaner-treated dogs would indicate a reduced contact time with the host as a result of acaricidal treatment.

## Methods

Three studies were conducted consisting of experimental tick infestation of dogs after single oral administration of fluralaner (Bravecto^TM^, MSD Animal Health) to dogs. Two studies were conducted with the European castor bean tick, *I. ricinus,* and one study was conducted with the American deer tick, also known as Blacklegged tick, *I. scapularis*. All studies included a treated group (Bravecto^TM^) and a negative-control group (untreated). Dogs in the Bravecto^TM^-treated group were weighed before treatment. The negative-control dogs remained either entirely untreated (*Studies 1* and *2*) or were only sham treated (*Study 3*). For inclusion in each study, dogs had to be clinically healthy, without ectoparasitic product treatment three months prior to study start, and were identified by microchip numbers. Each of the studies had a similar design for assessing tick vitality (i.e. ability of coordinated movement after thermal stimulation) and weighing of ticks, for which a calibrated analytical scale was used. The statistical analysis was performed using the software package SAS® (SAS Institute Inc., Cary, NC, USA, release 9.2).

### Studies 1 and 2

#### Study design

Four healthy Beagle dogs were clinically examined for inclusion in the study and assigned to two study groups (Bravecto^TM^ or negative-control group) of two dogs each. Dogs were group-housed indoors within their corresponding study group and fed a standard, commercially available dry dog food. In both studies dogs had free access to drinking water, and general health observations were performed once daily.

#### Infestation

Laboratory-reared ticks were used for repeated infestation of sedated dogs. Fifty unfed, adult female ticks were applied directly onto the fur, along the back, lateral side, and head of each dog. Approximately ten male ticks were additionally applied to provide *Ixodes* females with the best conditions for attachment and feeding to repletion. For the infestation process, sedated dogs (0.1 ml medetomidinhydrochlorid/kg bodyweight by intramuscular injection, e.g. Domitor®) were kept separately in an infestation box for up to 3 h. In *Study 1*, tick infestations with *I. ricinus* were performed at 7 days (week 1), 28 (±1) days (week 4), 56 days (week 8) and 84 days (week 12) after treatment. In *Study 2,* tick infestations with *I. scapularis* in the Bravecto^TM^-group were performed at 1 day after treatment (week 1), and dogs in the negative-control group were infested three days later (due to resource constraints). Further infestations were conducted in both study groups at 31 days (week 4), 56 days (week 8) and 84 days (week 12) after treatment.

#### Tick collection

Starting 1 day after the week 1 infestation, three female ticks per dog were randomly collected (if less than three ticks were present on dogs, the available number of ticks per dog was collected). Tick collection was continued daily until no more ticks were found. After the infestations at week 4, week 8 and week 12, all ticks were collected from dogs at once, approximately 48 h (*I. ricinus*) or approximately 12 h (*I. scapularis*) after the respective infestation.

#### Assessment

Collected female ticks were assessed for vitality (dead/alive) and each female tick was weighed to quantify the extent of fluralaner uptake since attachment. Thereafter, ticks were photographed to further illustrate tick development on treated versus untreated dogs. Additionally, separate sets of 20 unfed female ticks per species (*I. ricinus*, *I. scapularis*) were obtained from the same batch used for the infestations during the study (i.e. these adult ticks had not been used for host infestations). These ticks were weighed for comparison, and henceforth are referred to as baseline ticks.

#### Statistical analysis

All group comparisons were independent, testing for significant differences in tick weights. The statistical test applied was a two-sided, two-sample *t*-test. Depending on the results of a preceding equality of variance test, either pooled variances (equal variance situation) or the Satterthwaite approximation (unequal variance situation) were used for the *t*-test. The level of significance was set to α = 0.05.

In both studies, comparisons of tick weights from Bravecto^TM^-treated and negative- control groups were carried out for all assessment times, and additionally, comparisons of ticks collected from Bravecto^TM^-group and baseline ticks were carried out.

### Study 3

#### Study design

Ten healthy Beagle dogs (five males and five females) were used in the study. During a pre-treatment period, a tick infestation (*I. ricinus*) was conducted to evaluate the susceptibility of each dog to experimental infestation and for random allocation of the dogs to the study groups based on total counts of live ticks obtained two days after the infestation. Nine dogs with the highest tick counts were randomized to two study groups (Bravecto^TM^-group of six dogs; negative-control group of three dogs). In addition, veterinary examinations, general health observations, and the weighing of all animals were performed during the pre-treatment period.

#### Infestation

Ticks of a laboratory-reared *I. ricinus* isolate, which was regularly substituted with ticks caught from the field, were used for the infestations of sedated dogs (0.1 ml medetomidinhydrochlorid/kg bodyweight intramuscular, e.g. Domitor®). Dogs were infested with approximately 50 viable, adult, unfed female *I. ricinus* ticks. Approximately five male ticks were additionally applied to provide the *Ixodes* females with the best conditions for attachment and feeding to repletion. Infestations took place two days before treatment to assess immediate efficacy, and 28 days (4 weeks), 56 days (8 weeks) and 84 days (12 weeks) after treatment to assess persistent efficacy. For the infestation process and for sedation, dogs were kept separately in an infestation box for up to three hours, and were then housed individually until removal of ticks.

#### Tick collection

Tick removal/counts on each animal were carried out 48 (±2) hours after treatment to assess immediate efficacy, and 48 (±2) hours following each infestation at 4, 8 and 12 weeks to assess persistent efficacy.

#### Assessment

The study personnel who carried out tick assessments (i.e. vitality, weight and coxal index) were masked with regard to the treatment status of each dog. Live and dead ticks were counted and the intact ticks of both groups were weighed on each assessment day. Additionally, the scutal width across the dorsal side of the ticks and the gap between the bases of the fourth pair of legs across the ventral abdomen of the ticks were measured using a stereomicroscope (magnification of 25). The coxal index was then calculated as a ratio of coxal gap to scutal width [15, 16].

Furthermore, a separate set of 20 unfed female ticks was obtained from the same batch used for the respective infestation at each assessment time (i.e. adult ticks had not been infested to a dog). These ticks were also weighed for comparison and used as baseline ticks.

#### Statistical analysis

The statistical analysis of tick weight and coxal index was conducted as described in *Studies 1* and *2*. Adequacy of infestation was assessed on each tick count day by determining the mean tick attachment rate of live ticks in the control group. Efficacy for the fluralaner group was calculated at each assessment time point using geometric (or arithmetic) means with Abbott’s formula:

Efficacy (%) = 100 x (MC – MT)/MC, where MC was the mean number of total live attached ticks on the untreated dogs, and MT was the mean of total live attached ticks on the Bravecto^TM^-treated dogs. Log-transformed tick counts in both study groups were compared using a two –sample *t*-test. The two-sided level of significance was set to α = 0.05. Efficacy was claimed if the efficacy against ticks in the Bravecto^TM^-treated group was greater than or equal to 90 %.

## Results

All dogs (treated and control) were healthy at study enrollment, on day of treatment administration and throughout the duration of each study. At treatment all dogs swallowed the tablet and therefore were successfully dosed, and no adverse events were observed in any treated dog.

In all studies, the mean attachment rates on negative control dogs were at least 31 % at all times of assessment demonstrating that ticks used were vigorous.

**Fig. 1:**
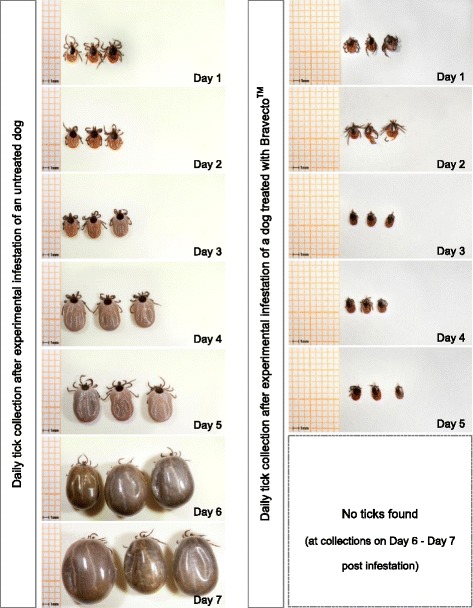
Example of *I. ricinus* - tick growth on an untreated dog (left) in comparison to ticks killed after infesting a dog treated with Bravecto^TM^ (right) (*Study 1*)

**Fig. 2:**
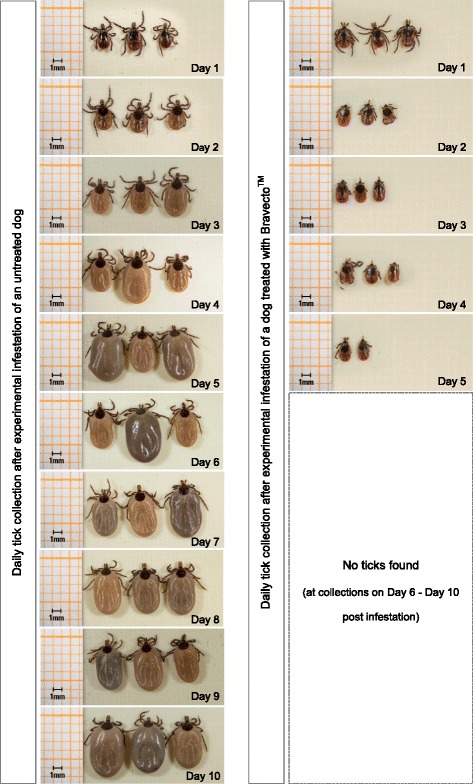
Example of *I. scapularis* - tick growth on an untreated dog (left) in comparison to ticks killed after infesting a dog treated with Bravecto^TM^ (right) (*Study 2*)

The mean tick weights of *Study 1* and *Study 2* are presented in Table 1 (*I. ricinus*) and Table 2 (*I. scapularis*), respectively. Photos of ticks collected daily from Bravecto^TM^-treated and negative-control dogs are presented in Fig. 1 (*I. ricinus*) and Fig. 2 (*I. scapularis*).

**Table 1:** Tick weights of *I. ricinus* collected from Bravecto^TM^-treated and untreated dogs in *Study 1*

Infestation	Collection	Tick weight^a^ [mg]					
		Bravecto^TM^			Negative control		
		Mean	Min	Max	Mean	Min	Max
Week 1	Daily after infestation	1.0^e^	0.7	1.9	2.2	1.8	3.0
		1.4^e^	0.7	2.5	4.2	3.6	5.2
		0.7^e^	0.5	1.3	8.4	6.2	10.2
		0.6^eb^	0.5	0.7	21.4	19.9	23.8
		0.4^ec^	0.3	0.6	33.2	24.9	39.1
		N/A	N/A	N/A	211.5	133.7	290.8
		N/A	N/A	N/A	186.9	22.4	402.4
		N/A	N/A	N/A	227.0^d^	179.9	300.8
Week 4	2 days after infestation	0.8^e^	0.4	2.3	3.9	2.8	6.1
Week 8		1.5^e^	0.7	3.0	4.1	2.0	8.4
Week 12		1.5^e^	0.5	2.8	4.3	3.1	9.4

*N/A* Not applicable because no ticks were found on dogs

^a^Arithmetic mean, minimum (Min) and maximum (Max) tick weight

^b, c, d^Less than three ticks per dog were present at tick collection. Therefore, the mean tick weight was derived from five (^b^) or three ticks (^c^) in the treated group, and from three ticks (^d^) in the negative-control group

^e^Tick weight in treated group is significantly different from negative control (*p* ≤ 0.0006)

**Table 2:** Tick weights of *I. scapularis* collected from Bravecto^TM^-treated and untreated dogs in *Study 2*

Infestation	Collection	Tick weight^a^ [mg]					
		Bravecto^TM^			Negative control		
		Mean	Min	Max	Mean	Min	Max
Week 1	Daily after infestation	1.0^e^	0.7	1.2	1.7	1.5	2.4
		0.9^e^	0.6	1.9	3.6	3.1	4.1
		0.7^e^	0.6	0.8	6.2	4.8	9.4
		0.6^e^	0.4	0.9	12.2	5.9	23.5
		0.6^eb^	0.4	0.8	22.8	11.4	37.5
		0.6^c^	0.6	0.6	17.8	10.1	38.7
		N/A	N/A	N/A	16.2	7.8	23.2
		N/A	N/A	N/A	23.1^d^	13.6	56.9^d^
		N/A	N/A	N/A	23.3	12.1	35.2
		N/A	N/A	N/A	21.1	11.7	30.7
Week 4	Approx.12 h after infestation	1.7	1.0	2.7	1.8	1.0	2.4
Week 8		1.6^e^	1.0	2.1	1.8	1.3	2.1
Week 12		1.6^e^	0.7	2.2	1.8	1.4	2.1

*N/A* Not applicable because no ticks were found on dogs

^a^Arithmetic mean, minimum (Min) and maximum (Max) tick weight

^b, c^Less than three ticks per dog were present at tick collection. Therefore, the mean tick weight was derived from four (^b^) or one tick (^c^) in the treated group

^c^No statistical analysis was performed because only one tick was recovered from treated group

^d^The mean tick weight was derived from seven ticks in the negative-control group because a fourth tick (^d^) dropped from one control dog after tick collection and was included in the assessment

^e^Tick weight in treated group is significantly different from negative control (p-values in the range of 0.0108 to < 0.0001)

For *Study 3* (*I. ricinus*) the mean tick weight results are presented in Table 3, the coxal indices in Table 4 and tick efficacy results are given in Table 5.

**Table 3:** Tick weights of *I. ricinus* collected from Bravecto^TM^-treated and untreated dogs in *Study 3*

Infestation	Collection	Tick weight [mg]			
		Bravecto^TM^		Negative control	
		Mean^a^	Stdev^b^	Mean^a^	Stdev^b^
Week 1	2 days after infestation	4.32^c^	3.51	10.88	5.85
Week 4		1.41^c^	1.31	3.80	0.59
Week 8		2.26^c^	1.82	3.65	0.72
Week 12		2.38^c^	1.72	3.76	0.59

^a^Arithmetic mean

^b^Standard deviation

^c^Tick weight in treated group is significantly different from negative control (*p* < 0.0001)

**Table 4:** Coxal indices of *I. ricinus* collected from Bravecto^TM^-treated and untreated dogs in *Study 3*

Infestation	Collection	Coxal index			
		Bravecto^TM^		Negative control	
		Mean^a^	Stdev^b^	Mean^a^	Stdev^b^
Week 1	2 days after infestation	0.80^c^	0.26	1.14	0.20
Week 4		0.49^c^	0.21	0.74	0.07
Week 8		0.58^c^	0.26	0.68	0.06
Week 12		0.59^c^	0.25	0.73	0.07

^a^Arithmetic mean

^b^Standard deviation

^c^Coxal index in the treated group is significantly different from negative control (*p* < 0.0001)

**Table 5:** Mean tick counts and tick-killing efficacy after Bravecto^TM^ treatment conducted in * Study 3*

Assessment time^a^	Mean tick counts^b^ [n] (control/treated)	Efficacy [%] (*I. ricinus*)
Week 1	34.2/0^c^ (34.3/0)	100 (100)
Week 4	40.9/0^c^ (41.0/0)	100 (100)
Week 8	38.6/0.2^c^ (38.7/0.3)	99.5 (99.1)
Week 12	38.5/2.1^c^ (38.7/2.2)	94.6 (94.4)

^a^Efficacy assessment either 48 (±2) hours after treatment (week 1) or 48 (±2) hours after re-infestation (week 4, 8 and 12)

^b^Geometric mean results; in parenthesis () the arithmetic mean results are given

^c^Log-transformed live tick count in the treated group is significantly different from negative control (*p* < 0.0001)

### Study 1: Oral treatment of fluralaner (Bravecto^TM^) against experimental infestation of *I. ricinus* on dogs

All ticks collected from Bravecto^TM^-treated dogs after infestation at week 1, week 4, week 8 and week 12 were dead, and all ticks collected from negative-control dogs were alive.

#### Tick weights

Baseline ticks weighed in the range of 1.3 to 2.1 mg, with a mean tick weight of 1.7 mg.

At all assessment times, the mean weights of ticks collected from Bravecto^TM^-treated dogs were significantly lower than the mean weights of ticks collected from negative-control dogs (p-values in the range of *p* = 0.0006 to *p* < 0.0001). The mean weights of ticks collected from Bravecto^TM^-treated dogs in week 1 and from week 4 to week 12 were significantly lower than those of baseline ticks (in both cases *p* < 0.0001).

### Study 2: Oral treatment of fluralaner (Bravecto^TM^) against experimental infestation of *I. scapularis* on dogs

All ticks collected from Bravecto^TM^-treated dogs were dead, and all ticks collected from negative-control dogs were alive.

#### Tick weights

Baseline ticks weighed in the range of 1.3 – 2.1 mg, with a mean tick weight of 1.7 mg.

At all assessment times, the mean weights of ticks collected from Bravecto^TM^-treated dogs were lower than the mean weights of ticks collected from negative-control dogs, and at all assessment times except at the 4-week assessment this difference in tick weight between both study groups was significant (*p* ≤ 0.0108). The mean weights of ticks from Bravecto^TM^-treated dogs at daily collections after the week 1 infestation were also significantly lower than those of baseline ticks (*p* < 0.0001).

### Study 3: Oral treatment of fluralaner (Bravecto^TM^) against experimental infestation of *I. ricinus* on dogs

At all infestation time points (including first infestation when the dogs’ ability to retain ticks was assessed), all control dogs were adequately infested (infestation rate of at least 25 % of the number of applied ticks), and therefore the efficacy calculation is considered valid.

#### Efficacy

Based on geometric (and arithmetic) means, the immediate efficacy against an existing tick infestation after Bravecto^TM^ treatment was 100 % (100 %), and persistent efficacy against tick challenges at 4, 8 and 12 weeks following Bravecto^TM^ treatment was 100 % (100 %), 99.5 % (99.1 %) and 94.6 % (94.4 %), respectively. At all assessment times, log-transformed live-attached tick counts in the treated group differed significantly from the log-transformed live-attached tick counts in the negative-control group (*p* ≤ 0.0001).

#### Tick weights

Baseline ticks had a mean tick weight of 1.99 ± 0.23 mg.

At all assessment times, the mean weights of ticks collected from Bravecto^TM^-treated dogs were significantly lower than the mean weights of ticks collected from the negative-control dogs (*p* < 0.0001). The mean weight of ticks collected from Bravecto^TM^-treated dogs after the 4-week infestation was also significantly lower than that of baseline ticks (*p* < 0.0144).

#### Coxal index

At all assessment times, the mean coxal indices of ticks collected from Bravecto^TM^-treated dogs were significantly lower than the mean coxal indices of ticks collected from negative-control dogs (*p* < 0.0001).

## Discussion

All studies demonstrated a consistent and high acaricidal efficacy and favorable tolerability after administration of Bravecto^TM^ to dogs. Twelve weeks of efficacy against ticks of *Ixodes* spp. was demonstrated in accordance with Bravecto^TM^ chewable tablets’ label claims [3], irrespective of the tick species tested (*I. ricinus*, *I. scapularis*). Even short-term tick exposure of approximately 12 h to Bravecto^TM^-treated dogs resulted in a potent tick-killing effect (*I. scapularis*), a finding that supports the 12-h speed of kill claim for ticks [3, 8].

To enable tick weight comparisons and allow for statistical analysis with sufficient numbers of ticks in treated and untreated groups, dogs in all studies were kept under controlled laboratory conditions which included avoidance of any potential influence that could cause ticks to detach during the infestation periods. In contrast to the home environment wherein dogs are often encouraged to be active (e.g. at dog walks, play time) and routinely experience intense human-dog and dog-dog interactions, the controlled study conditions restricted the handling of dogs to a minimum during the times when ticks were present. It can be assumed that this reduced the number of detaching ticks (particularly of dead ticks on treated dogs) and thereby enabled a statistical analysis of tick weights in both groups.

In line with the high consistency of acaricidal effect (i.e. tick efficacy) observed in the studies, the tick weight evaluations also demonstrated highly consistent results. The mean weights of ticks that were all killed when collected from Bravecto^TM^-treated dogs were lower than the mean weights of ticks from negative-control dogs, statistical comparison shows a significant difference at all assessment times except at week 4 (*Study 2*). The significant difference was demonstrated over the entire duration of tick efficacy (i.e. over 12 weeks, *p* ≤ 0.0108) and for both *Ixodes* species tested. Thus, the tick weight results of all three studies provide strong evidence that ticks encountering Bravecto^TM^-treated dogs are killed very quickly after attachment, and that the uptake of fluralaner before ticks start dying is minimal. This conclusion is substantiated by the coxal indices (*Study 3*), which were significantly lower in Bravecto^TM^-treated dogs compared to negative controls consistently over a 12 week period (*p* < 0.0001).

These data add to fluralaner’s demonstrated rapid killing effect [8] and suggest that Bravecto^TM^ is able to reduce the risk of tick-borne disease transmission in treated dogs. A recent transmission study further supports the conclusion and demonstrates a 100 % preventive effect over a 12-week period against *Babesia canis* transmission using infected *Dermacentor reticulatus* for repeated infestations of fluralaner (Bravecto^TM^) -treated dogs in comparison to negative control dogs [17].

The baseline ticks in the first two studies had mean tick weights of 1.7 mg (*I. ricinus*, *I. scapularis*) whereas in a third study the mean tick weight of baseline-*I. ricinus* was slightly higher (2.0 mg). Surprisingly, on some occasions the mean weights of ticks obtained from Bravecto^TM^-treated dogs were significantly lower than those of baseline ticks, which had not been infested to dogs (*p* ≤ 0.0162). This can be explained with a drying of ticks after being killed on treated dogs. The data support the very low uptake by ticks following systemic treatment of dogs that is further illustrated by the tick photographs in Figs. 1 and 2.

## Conclusions

Systemic treatment using oral administration of fluralaner (Bravecto^TM^) effectively controls tick infestations on dogs. The tick weights and coxal indices determined in these studies confirm that a minimal uptake results in sufficient exposure to fluralaner to induce a potent acaricidal effect in treated dogs.

### Compliance statement

The studies were in compliance with German animal welfare regulations, and ethical approval was obtained before start of each study. *Studies 1* and *2* were negative controlled studies conducted in Germany as non-GLP studies in a GLP compliant facility. *Study 3* was conducted in accordance with Good Clinical Practice (VICH guideline GL9, Good Clinical Practice (EMA, 2000)), as partially blinded, randomized, negative controlled efficacy study.
